# Enhanced Biomechanically Mediated “Phagocytosis” in Detached Tumor Cells

**DOI:** 10.3390/biomedicines9080947

**Published:** 2021-08-02

**Authors:** Yoel Goldstein, Katerina Tischenko, Yifat Brill-Karniely, Ofra Benny

**Affiliations:** Institute for Drug Research, The School of Pharmacy, Faculty of Medicine, The Hebrew University of Jerusalem, Jerusalem 9112001, Israel; yoel.goldstein@mail.huji.ac.il (Y.G.); katerina.tischenko@mail.huji.ac.il (K.T.); yifat.brill@mail.huji.ac.il (Y.B.-K.)

**Keywords:** 3D printing, particle uptake, cancer, cell mechanics, floating cells

## Abstract

Uptake of particles by cells involves various natural mechanisms that are essential for their biological functions. The same mechanisms are used in the engulfment of synthetic colloidal drug carriers, while the extent of the uptake affects the biological performance and selectivity. Thus far, little is known regarding the effect of external biomechanical stimuli on the capacity of the cells to uptake nano and micro carriers. This is relevant for anchorage-dependent cells that have detached from surfaces or for cells that travel in the body such as tumor cells, immune cells and various circulating stem cells. In this study, we hypothesize that cellular deformability is a crucial physical effector for the successful execution of the phagocytosis-like uptake in cancer cells. To test this assumption, we develop a well-controlled tunable method to compare the uptake of inert particles by cancer cells in adherent and non-adherent conditions. We introduce a self-designed 3D-printed apparatus, which enables constant stirring while facilitating a floating environment for cell incubation. We reveal a mechanically mediated phagocytosis-like behavior in various cancer cells, that was dramatically enhance in the detached cell state. Our findings emphasize the importance of including proper biomechanical cues to reliably mimic certain physiological scenarios. Beyond that, we offer a cost-effective accessible research tool to study mixed cultures for both adherent and non-adherent cells.

## 1. Introduction

Interactions of cells with particles are essential for various biological processes, including pathogens neutralization, the elimination of cell debris and molecular signaling [1,2,3]. Synthetic particulate systems in the form of nano and micro carriers are widely used in drug delivery and targeted therapy. Measuring interactions of particle formulations with cells and tissues is essential for the performance assessment of drug vehicles, and for their optimization. Since carriers can contain active compounds, the issue of their uptake by cells is critical for the safety and efficacy of the treatment [4,5,6,7] and for drug targeting [8].

Cellular mechanisms of particle uptake are widely classified into passive and active uptake. Passive uptake commonly refers to mechanisms that do not require cell energy, such as in the transport against a gradient concentration. In contrast, an active cell uptake requires the investment of cellular energy in the process, as occurs in endocytosis [9]. These processes entail encapsulation of foreign bodies in vesicles created by cell membrane folding. In particles below ~200 nm, endocytosis is the main mechanism of internalization, while for larger objects, micropinocytosis and phagocytosis are the dominant mechanisms [10], which can be found in several types of cells, including designated phagocytic cells such as monocytes, macrophages, neutrophils and non-professional phagocytes, such as epithelial cells and fibroblasts [10,11,12,13]. Cancer cells were found to use phagocytic-like uptake very extensively, in correlation with cell deformability and malignancy, as discussed in detailed in our recent study [14]. During their “phagocytosis”, the cell body distorts and undergoes deformation. The uptake process of sub-micron particles, above the size that can be internalized by endocytosis, necessitates a physical contact and binding of the particles with cell membrane, which drives active particle engulfment [15,16]. The mechanical deformation of cells results from the combination of several parameters, such as the membrane elasticity and fluidity, cytoskeleton rigidity and dynamical rebuilding of the cytoskeleton [17,18,19,20,21,22,23,24,25,26,27]. These features control the ability of cells to modify their shape in different length scales, upon external or internal cues.

Commonly used in vitro assays for cell uptake have a limited capacity to mimic changing physiological scenarios. While cell uptake in adherent conditions is relevant for cells that reside in tissues, when cells shift into non-adherent conditions, for example in the case of circulating tumor cells, metastatic cells, hematological tumors (e.g., lymphomas and leukemia) and immune cells, it should be measured in floating conditions. Furthermore, critical issues in drug delivery, such as bio-distribution and performance of particulate systems, could be substantially affected by the biomechanical environment of the target cells.

Here, based on our previous work [14], we aim to examine the role of cell deformability, as a critical factor for phagocytosis-like particle uptake in cancer cells. Our hypothesis is that cell uptake in a two-dimensional static system may differ from detached conditions where the cell’s ability to deform is maximal. Floating conditions may better simulate certain physiological scenarios in vivo, especially of circulating cells.

To provide a useful tool for the systematic investigation of cell uptake in a floating versus adherent state, we designed and 3D-printed a continuous flow system (3DCFS). Compatible with standard lab equipment, the 3DCFS enables constant stirring while facilitating a controlled and continually floating environment for cell incubation. Using the 3DCFS, we fine-tuned the stirring velocity to maintain uniform mixing of cells and particles, while avoiding damage to the floating cells by excessive shear forces. In a set of experiments, we compared the uptake of particles by cancer cells in flowing conditions versus adherent conditions. The latter is the favored state for long-term viability and growth of the tested cancer cells. For the first 9 h of incubation, the non-adherent cells maintained high viability levels and showed significantly higher levels of particle uptake compared to cells spread in semi-2D conditions. This result is somewhat surprising since most of the physical characteristics of the two systems favor uptake in the adherent 2D rather than the non-adherent 3D system. However, our findings suggest that the higher deformability of the floating cells compared to the adherent ones confer a strong advantage to contributes to a fast particle uptake in the suspended scenario.

Our study demonstrates the significant role of mechanical cues on particle uptake by cells and the potential differences in various organs. Moreover, the mechanical state of target cells should be accounted for when designing optimal drug carriers and, thus, tested ex vivo in relevant conditions. For the field of drug delivery these important considerations may implicate for both the biodistribution and tissue absorption of colloidal systems and for the designing of more specific carriers. Finally, the methodology detailed here may provide a highly controlled, rapid and high-throughput assay for particle uptake with physiological relevance. The method may be used as a faster assay for particle uptake in adherent cells using their floating conditions, while considering the expeditious process.

## 2. Materials and Methods

### 2.1. 3D Printing

#### 2.1.1. 3D printer and Software

All objects were designed with AutoCAD^®^ (version 2018.3, Autodesk Inc, San Rafael, CA, USA) saved in their final form in STL format and uploaded to Asiga composer (version 1.2, Asiga, Sydney, NSW, Australia). Luxaprint^®^ mould Clear resin (DETAX, Ettlingen, Germany) (wavelength 385 nm), which is utilized for the generative manufacturing of hard ear molds and hearing protection, was used in this study for printing 3D molds. We printed with the DLP-SL (digital-light-processing stereolithography) printer Asiga Max-X27 UV (Sydney, NSW, Australia). This 3D-printer has aa LED light source with 385 nm UV wavelength. The XY pixel resolution of the printer’s projectors was 27 µm and its minimum Z plane resolution was 1 µm. The maximum build size X, Y and Z was 51.8 × 29.2 × 75 mm, respectively.

#### 2.1.2. 3D Printing Procedure

Before starting, the vat was filled with the resin and positioned in the printer under the build plate. Then, the build plate was lowered into the vat to a predetermined height, and the DLP projected the first slice of the design for a predetermined amount of time. Next, the build plate rose for a few seconds and then returned to the vat, and the DLP projected the next slice of the design. Subsequently, the build plate rose again, and this process continued until the entire design was printed. Next, the printed object was removed from the build plate, rinsed with isopropyl for 3 min in a sonicator bath (BANDELIN, Germany), dried using air pressure and cured in a UV oven for 5 min (PCU LED, Dreve, Germany).

### 2.2. Cell Culture

Experiments were performed using A375 (primary human melanoma cells), MDA-MB 231 (human breast adenocarcinoma), BXPC-3 and AsPC-1 (both human pancreas adenocarcinoma cells) cells. All cell lines are originated from ATTC (American Type Culture Collection, Manassas, VA, USA) and were mycoplasma-free. Prior to the experiments, the cells were seeded and incubated to 70–80% confluence on a 10 cm dish. A375 and MDA-MB 231 cells were seeded and incubated in Dulbecco’s modified Eagle’s medium (DMEM, Sigma Aldrich, Darmstadt, Germany), and supplemented with 10% (*v/v*) fetal bovine serum, 1% antibiotic (streptomycin (10,000 µg/mL) and penicillin (10,000 units/mL)) at 37 °C with 5% CO_2_, trypsinized and counted. BXPC-3 and AsPC-1 cells were seeded and incubated to 70–80% confluence prior to experiments in RPMI-1640 medium (Sigma Aldrich, Darmstadt, Germany), that was supplemented with 2 mM L-glutamine, 10 mM HEPES, 1 mM sodium pyruvate, 4500 mg/L glucose and 1500 mg/L sodium bicarbonate, at 37 °C with 5% CO_2_.

### 2.3. Viability Assay

10 mL DMEM of media was inserted in the 3DCFS device and 6 × 10^6^ A375 cells were added. The 3DCFS was then incubated at 37 °C with 5% CO_2_ on a stirrer (IKA^®^ Color Squid, Sigma Aldrich, Germany) at 150 rpm. Two sets of samples were taken before the cells were exposed to the 3DCFS: unstained control and stained cells after 5 min (time 0). Once in 3DCFS, the cells were sampled (300 µL) after 3, 6, 9, and 24 h, 300 µL per sample hours. All the samples were centrifuged at 1200 rpm for 5 min, washed with phosphate-buffered saline (PBS), and stained with calcein AM (Cayman Chemical, Ann Arbor, MI, USA) for 30 min at room temperature (dilute aliquot 1:1000). The results were normalized to the viability control samples taken at time 0.

For testing the recovery of cells in floating conditions, 10 mL RPMI medium was added to the 3DCFS and 7 × 10^6^ BXPC-3 cells were inserted and incubated at 150 rpm for 4 h. Then, 100 µL samples were taken after 1, 2, 3 and 4 h, seeded in 6-well plate wells with 3 mL media and incubated for 48 h. Similar protocols were used to assess cell viability post 2 mM Cisplatin treatment as detailed below.

### 2.4. Apoptosis Assay

Identification of apoptotic and necrotic A375 cells was performed and analyzed using BD LSRFortessa^TM^ flow cytometer (BD, San Jose, CA, USA) and FlowJo software using FITC Annexin V Apoptosis Detection Kit with PI (BioLegend, San Diego, CA, USA). A total of 300,000 cells were seeded in 6-well plate wells with 3 mL media for 24 h of incubation. Next, the 10 × 10^6^ cells were added to 10 mL media in the 3DCFS and incubated at 37 °C with 5% CO_2_ on a stirrer at 150 rpm for 24 h. Then, the cells were washed twice with cold cell-stained buffer and resuspended in Annexin V binding buffer at concentration of 1.0 × 10^6^ cells/mL. An amount of 100 µL of the suspended cells was transferred to a 5 mL tube, then 5 µL of FITC Annexin V and 10 µL of PI solution were inserted, gently vortexed and incubated for 15 min at room temperature in the dark. Then, 400 µL of Annexin V binding buffer was added. The positive control cells were exposed to 55 °C for 20 min.

### 2.5. Particles

In all studies mentioned, we used 1% (*w*/*v*) 0.8 µm purple (Ex. 580nm, Em. 620/30 nm) Polystyrene beads (Spherotech Inc., Lake Forest, IL, USA).

### 2.6. Uptake Assay

#### 2.6.1. Uptake Assay for Adherent Cells

A total of 300,000 A375 cells were seeded in 6-well plate wells with 3 mL media and were allowed to adhere overnight at 37 °C with 5% CO_2_. At this point, samples of cells without particles were taken as unstained controls. Then, particles were added to the plate wells (1 µL/1 mL) and incubated at 37 °C with 5% CO_2_ for up to 9 h. Afterwards, the samples were rinsed twice with cold PBS, trypsinized, centrifuged at 1200 rpm for 5 min at 4 °C and washed with PBS.

#### 2.6.2. Uptake Assay for Floating Cells

10 mL media were inserted in the 3DCFS and 3 × 10^6^ cells were added. Particles were added to the 3DCFS (1 µL/1 mL) and then incubated at 37 °C with 5% CO_2_ on a stirrer at 150 rpm for up to 9 h. Afterwards, 300 µL samples were taken, centrifuged at 1200 rpm for 5 min at 4 °C and washed with PBS.

### 2.7. Measurement of Particle Uptake by Cells

After incubation with particles, the cells were washed with cold PBS, detached using trypsin, washed again and filtered through a 4 to 50 mm nylon mesh using a 50 mL conical tube to remove tissue debris mesh. Cells were then centrifuged and suspended in a FACS buffer containing 1% bovine serum albumin in PBS and 0.05% sodium azide. A total of 10,000 events were acquired from each sample and analyzed using a BD LSRFortessa^TM^ flow cytometer and FlowJo software (BD, San Jose, CA, USA). The unstained samples were gated prior to the assay measurements.

### 2.8. Validation of 3DCFS Performance

#### 2.8.1. Mixing Uniformity Measurement in 3DCFS

In order to ensure uniformity of mixing in 3DCFS container, we measured particle uptake levels in various fluid planes. A total of 10 × 10^6^ A375 cells were added to 10 mL DMEM for incubation in the 3DCFS stirred at 150 rpm. Particles (1 µL/1 mL) were added immediately after to the 3DCFS. Three levels of sampling from: the bottom, middle, and top of the 3DCFS were compared. A total of 300 µL per sample were taken, centrifuged at 1200 rpm for 5 min at 4 °C, and washed with PBS for each sampling level after 2, 4, 6 and 24 h. For each time interval, the average uptake level was normalized to the average uptake level of the top sample.

#### 2.8.2. 3DCFS Blade Directionality

Particle uptake by cells was measured in adherent and non-adherent conditions after 2, 4, 6 and 24 h of incubation. A total of 300,000 A375 cells were seeded in 6-well plate wells with 3 mL media and were allowed to adhere overnight at 37 °C with 5% CO_2_. Simultaneously, a total of 10 × 10^6^ A375 cells were added to 10 mL DMEM for incubation in two 3DCFS devices using opposing blade direction (clockwise and counterclockwise) on a stirrer at 150 rpm. Immediately after, particles were added to both devices (1 µL/1 mL). Adherent samples were rinsed twice with cold PBS, trypsinized, centrifuged at 1200 rpm for 5 min at 4 °C and washed with PBS. For the 3DCFS system, 300 µL samples were taken, centrifuged at 1200 rpm for 5 min at 4 °C and washed with PBS.

### 2.9. Measurement of Cell Division

Cells in adherent conditions were counted as follows: a total of 300,000 A375 cells were seeded in 6-well plates with 3 mL media and were allowed to adhere for 24 h at 37 °C with 5% CO_2_. The cells were then trypsinized, centrifuged and counted using a Countess automated cell counter (Thermo Fisher Scientific, Invitrogen, Waltham, MA, USA). For the floating conditions, cell number was determined over time; 10 mL media were added to the 3DCFS followed by the addition of 10 × 10^6^ suspension cells. A total of 100 µL was sampled after 2, 4, 6, 8 and 24 h and was counted using the Countess Automated Cell Counter.

### 2.10. F-Actin Cell Staining

#### 2.10.1. Adherent Conditions

To detect F-actin filament in cells grown in different attachment conditions, A375 cells were grown in 6-well plates on a cover slip with 3 mL DMEM media, and were allowed to adhere for 24 h at 37 °C with 5% CO_2_. The cells were then fixed with 4% paraformaldehyde for 15 min, rinsed three times with 1× PBS, permeabilized with 0.1% Triton-X for 10 min and rinsed three times with 1× PBS. Alexa Fluor^®^ 555 phalloidin Ex/Em 555/565 nm (Invitrogen, Waltham, MA, USA) was applied according to the manufacturer’s instructions. Nuclei were visualized with 4′,6-diamidino-2-phenylindole (DAPI) stain in a 1 μg/mL solution. The cover slip was mounted using Fluoromount Aqueous Mounting Medium (Sigma-Aldrich, Germany) according to the manufacturer’s instructions. Images were obtained using confocal microscopy (Nikon’s A1 MP multiphoton confocal microscope equipped with a 639 nm diode, New York, NY, USA) and analyzed using NIS-Elements.

#### 2.10.2. Floating Conditions

A total of 10 × 10^6^ A375 suspension cells were incubated in the 3DCFS with 10 mL DMEM media at 37 °C, with 5% CO_2_ on a stirrer at 150 rpm. Then, 500 µL samples were taken after 2 and 4 h of incubation, centrifuged at 1200 rpm for 3 min, fixed with 100 µL 4% paraformaldehyde for 3 min, rinsed once with 100 µL PBS and permeabilized with 100 µL 0.1% Triton-X for 2 min, followed by another washing step. Alexa Fluor^®^ 555 phalloidin Ex/Em 555/565 nm (Invitrogen, Waltham, MA, USA) was applied according to the manufacturer’s instructions. The samples were then stained with DAPI 1 μg/mL concentration and washed again. Then, 20 μL of cells were sealed and mounted using Fluoromount Aqueous Mounting Medium (Sigma-Aldrich, Germany) according to the manufacturer’s instructions, between cover slip and a glass slide. Images were obtained using confocal microscopy (Nikon’s A1 MP multiphoton confocal microscope equipped with a 639 nm diode) and analyzed using NIS-Elements.

### 2.11. Cell Viability and Particle Uptake Measurements of Post Cell Stiffness Modification with Cisplatin

#### 2.11.1. Viability Assay

For 96-well plate: Cell viability was measured using a colorimetric assay for 96-well plates with 2-(4-iodophenyl)-3-(4-nitrophenyl)-5-(2,4-disulfophenyl)-2H-tetrazolium monosodium salt (WST-1) reagent (Cayman Chemical, Ann Arbor, MI, USA). A WST-1 mixture was prepared according to the manufacturer’s instructions. A375 cells were seeded and incubated to reach 80% confluence in a 96-well plate dish in 100 µL medium and were allowed to adhere overnight at 37 °C with 5% CO_2._ Then, the cisplatin stock was diluted in medium to reach concentrations of 0.1, 0.5, 1, 10 and 100 µM, and they were added to the cells for 24 h of incubation at 37 °C with 5% CO. After incubation, 10 µL of WST-1 mixture was added to each well and then the plate was incubated for an additional 1.5 h. Cell viability was measured at 450 nm in a microplate reader (Spark 10M, Tecan, Männedorf, Switzerland).

For 6-well plate and 3DCFS: The cells’ viability was measured using calcein AM as described (Section 2.3)

#### 2.11.2. Particle Uptake

For 96-well plate: A375 cells were seeded and incubated to reach 80% confluence in 96-wells plate dish in 100 µL medium and were allowed to adhere overnight at 37 °C with 5% CO_2_. Then, the cisplatin stock was diluted in medium to reach concentrations of 1, 2, 5 and 10 µM and added to the cells for an additional 6 h of incubation at 37 °C with 5% CO_2_. Particle uptake levels were measured at 450 nm in the same microplate reader (Tecan spark 10M).

For 6-well plate and 3DCFS: After cisplatin treatment, uptake assays were performed as detailed above in both adherent and floating conditions. (Section 2.6).

## 3. Results

### 3.1. Design and 3D Printing of Apparatus for Cell Uptake Assays

**Figure 1:**
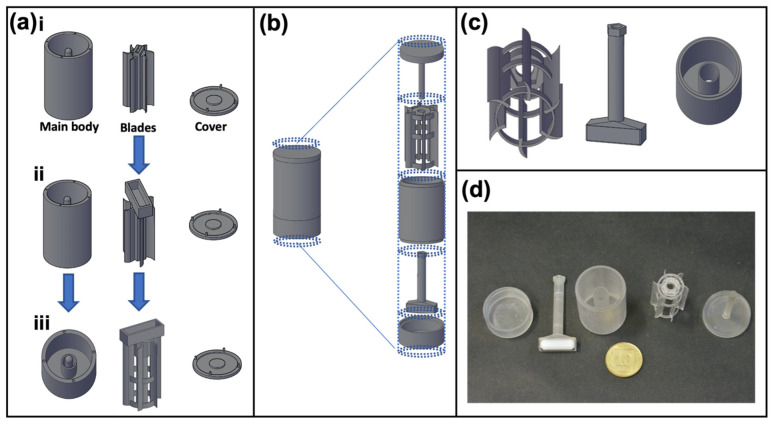
Evolution of the 3DCFS design. The purpose of the 3DCFS is to obtain a continuous and uniform stirring of cells and medium over time. (**a**,**b**) Changes made to the design (depicted in AutoCAD). (**a.i**) “Magnet above” includes three parts: main body with a central hinge, blades with magnet pocket and cover. (**a.ii**) Enlarging the magnet pocket. (**a.iii**) Adding an internal cylinder and reshaping the blades to prevent fluids from wetting the central pole and interfering with the blades’ movements. “Magnet below” includes five parts (top to bottom): (**b**,**c**) cover with a central hinge, blades, main body, magnet holder and a bas, that all fit together and move as one unit. (**d**) Photograph of the final 3D printed 3DCFS parts.

In order to perform controlled particle uptake studies in floating conditions compared to traditional adherence measurements, we designed the 3DCFS as a device compatible to a standard laboratory stirrer that was utilized to generate motion in a rotor. The latter would be centered in a main body container of 10 mL media and maintain cells’ viability and optionally (possibly) other components under constant motion. The device underwent four iterations before reaching a satisfactory final design (Figure 1a,b).

To keep the design simple and accessible, a standard magnet stirrer (IKA Color Squid) served as the engine and a magnet bar was placed so that the 3DCFS’s blades would spin with the magnet bar movement. Our initial approach was to place the magnet bar on top of the blades (Figure 1a.i), but despite different modification and adjustments (SI-methods, Figure 1a.i–a.iii), we failed to achieve an active and continuous spinning of the blades. When incubated, the 3DCFS’s parts accumulated humidity, increasing the friction between them and causing the blades to stop spinning. To tackle this problem, we decided to radically change our approach by placing the magnet beneath the blades. This was a challenge because it was important to separate the magnet from the fluid compartment to prevent damage to the cells. To achieve a robust and simple operation, we separated the magnet from the blades.

The final design included a container with a separate chamber for the magnet, a hollow magnet holder, blades and a cover (Figure 1b,c). When assembled, the magnet holder passed through the container and connected to the blades (Figure 1d). The cover was comprised of a central hinge that aligned with the hollow magnet holder; thus, stabilizing the device. This time, the 3DCFS blades continued spinning for hours under incubation.

### 3.2. Cell Death and Viability

**Figure 2:**
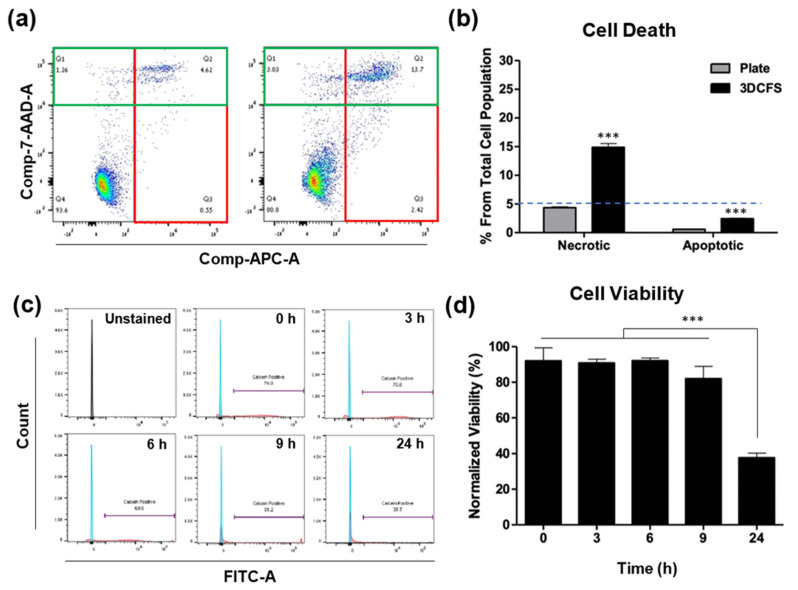
Identification of apoptosis, necrosis and cell viability. A375 cells were seeded and incubated both in static and floating condition for 24 h and underwent apoptosis assay using Annexin V and PI staining. (**a**) FACS’s scatter plot of cells in static conditions (left) and in floating conditions (right). The Annexin and PI positive cells are marked in green and red rectangles, respectively. (**b**) The results show that the percentage of both apoptotic and necrotic cells in static condition was less than 5%, while in floating condition, the percentage of apoptotic cells was under 5% and of necrotic cells approximately 15%. (**c**,**d**) Viability of A375 cells in the 3DCFS was measured at different time points over 24 h. (**c**) Cells were sampled at different labeled intervals, treated with calcein AM and underwent FACS analysis. (**d**) The results were normalized to a control measurement and show that the A375 cells kept their viability levels for as long as 9 h floating in the 3DCFS and decreased by approximately 50% after 25 h. *p*-value: *** ≤0.001.

We aimed to define the time regime at which reliable uptake experiments can be performed using the 3DCFS, while ensuring a high viability of the tested cells. A possible reduction in cell viability may result from the anoikis, but also from the shear or from potential toxicity of the 3D printing resin. A375 cells were used and cell death was analyzed using flow cytometry following Annexin V/PI staining. We found that, after 24 h, only <5% of the floating cells underwent apoptosis and approximately 15% underwent necrosis (Figure 2a). Additionally, staining with calcein AM to ensure a viable culture before stirring demonstrated that during the first 6 h in floating conditions, the cells maintained their high (~90%) viability as in time 0. After 9 h, a small decrease in cell viability was observed (<10% reduction); after 24 h, cell viability was dramatically decreased, leaving an average of 40% live cells (Figure 2b). The proliferation of cells in both conditions was measured over 24 h showing that cells grown on the plate underwent cell division and increased 2.3 times in number (Figure S1a), whereas the number of floating cells stayed constant during the first 8 h and then decreased by more than 50% after 24 h (Figure S1b), in agreement with our viability assay results. Following our observation, we fixed time frames for uptake experiments to 24 h incubation for adherent conditions and up to 9-hou’ incubation for floating conditions.

### 3.3. Particle Uptake

**Figure 3:**
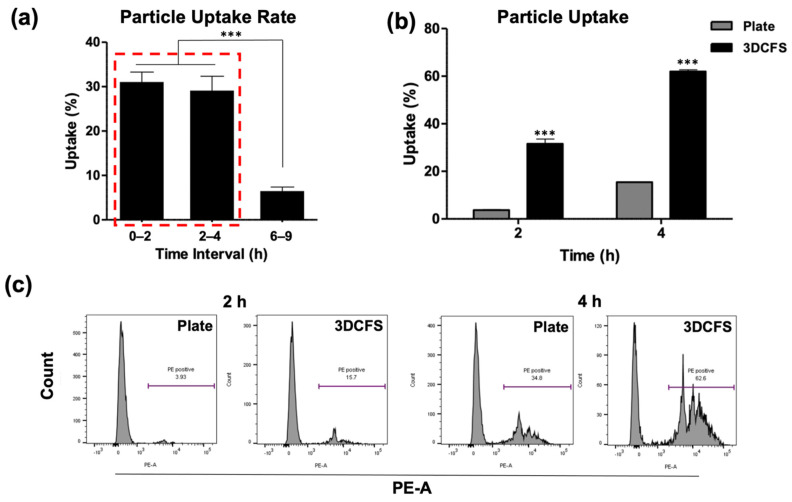
Uptake analysis of A375. A375 cells’ uptake capability was evaluated both when seeded on a plate and floating in the 3DCFS. (**a**–**c**) 0.8 µm particles were added to the systems and were sampled and analyzed by FACS up to 9 h of incubation. (**a**) Cell particle uptake percentage levels in the 3DCFS during the first 9 h of incubation. Cell particle uptake capability decreased significantly after 6 h compared to the first 4 h (red rectangle). (**b**) Cell particle uptake capability was significantly higher in the 3DCFS compared to the plate after 2 and 4 h of incubation. (**c**) The effect of additional time on the cells’ particle uptake capability was tested. The FACS analysis showed that the cells’ particle uptake capability increased when given more time after 2 h, both on the plate (left) and in the 3DCFS (right). *p*-value: *** ≤ 0.001.

The uptake of fluorescently labeled polystyrene particles at the size of 0.8 µm by A375 cells was measured. Unstained cells at time 0 were used for gating the FACS measurements to detect the positive cells that engulfed particles. After particle supplement, the 3DCFS was placed and incubated on a stirrer at 150 rpm and uptake levels were calculated for intervals. Between 0 to 2 h and 2 to 4 h of incubation, the uptake level in the 3DCFS was similar—approximately 30%. However, between 6 and 9 h, the uptake percentage dropped dramatically to approximately 7% (Figure 3a). Subsequently, our studies focused on the first 4 h, enabling us to compare particle uptake levels between both systems while eliminating the effect on viability. FACS analysis showed a clear and significant difference between the two mechanical conditions (Figure 3b,c); during the first 2 h, the floating cells exhibited an ~ eight-fold higher particle uptake than under the 2D conditions and ~ four-fold more after 4 h. After 4 h of incubation, the adherent cells’ average particle uptake reached 15%, while the floating cells’ average particle uptake reached more than 60%. In both conditions, the uptake approximately doubled between the second and fourth hour of incubation (Figure 3b). To eliminate the effect of cell confluence under adherent conditions (to avoid the settling of particles directly on the plate with no contact with the cells), we seeded A375 cells to reach 80% and 100% confluence, followed by 4 h of incubation with particles. In both cell densities there was ~10% difference in uptake, but there was a significantly lower uptake compared with an equivalent cell suspension in 3DCFS system, suggesting a marginal effect of particle absorbance in plastic (Figure S2).

**Figure 4:**
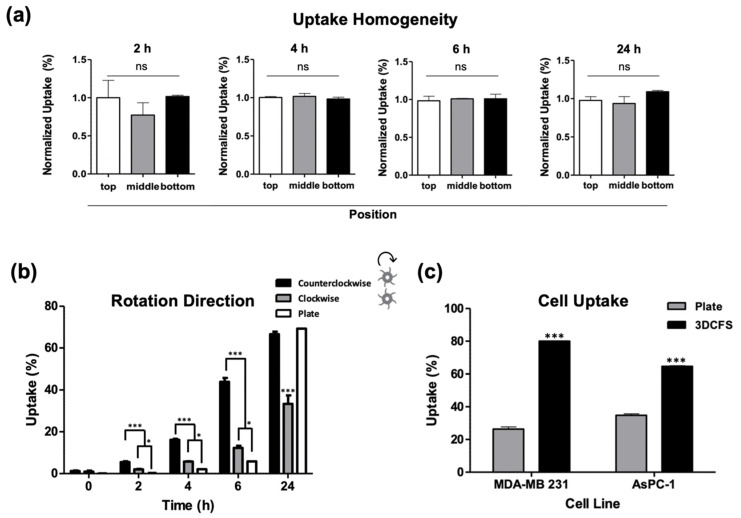
Validation of the 3DCFS performance. (**a**) Cells were sampled from the 3DCFS’s bottom, middle and top levels after 2, 4, 6 and 24 h of incubation to measure the homogeneity of their particle uptake levels. For each of the time points, there was no significant difference between the samples. (**b**) Uptake levels were compared between rotation directions in floating conditions and with the uptake level in adherent conditions. (**c**) Uptake capability of MDA-MB 231 and AsPC-1 cells was also evaluated both when seeded on a plate and in the 3DCFS. Both cells showed significant increase in the capability of the cells to uptake the particles after 6 h of incubation in the 3DCFS compared to the plate. *p*-value: *** ≤0.001, * ≤0.05, ns >0.05.

To ensure uniformity in the mixing conditions, cells were sampled from different locations in the chamber, i.e., from the bottom, middle, and top compartments of the 3DFCS’s main chamber and at different time points, which confirmed comparable uptake levels (Figure 4a). Since the design of the 3DCFS blades was curved, rotation in the clockwise direction (blades are concave) created a gripping movement while rotation in the counterclockwise direction (blades are convex) created a wavy movement. Therefore, we examined the particle uptake levels under both directions and found that it had a great impact (Figure 4b). Although the uptake showed similar trends in both systems, the proportion of uptake significantly favored the counterclockwise direction. During the first 6 h, both 3DCFS stirring directions yielded results superior to the 2D system. However, after 24 h, the uptake level of the 2D system reached that of the 3D system being stirred in the counterclockwise direction; whereas when the 3D system was stirred in the clockwise direction, it only reached ~50% the level of the 2D system (Figure 4b).

To verify that our results were not specific to one cell line only (A375) we measured particle uptake also in additional cancer cell lines: MDA-MB 231 (human breast adenocarcinoma) and AsPC-1 (human pancreas adenocarcinoma) cells, confirming similar uptake profiles and trends (Figure 4c).

### 3.4. Mechanical Modifications and Cellular Effects

**Figure 5:**
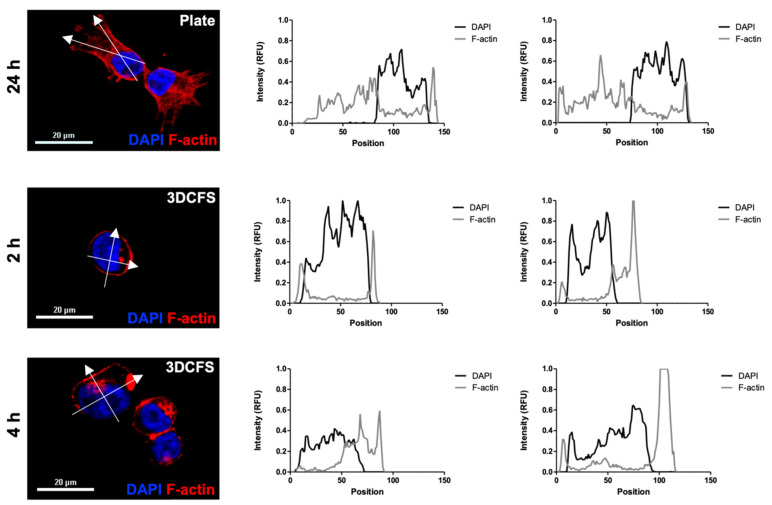
A375 F-actin density. Actin filaments of A375 cells were stained using rhodamine phalloidin and their intensity profile was measured at relevant time points for each system. (**a**) 24 h incubation in adherent conditions; (**b**) 2 h incubation in the 3DCFS; (**c**) 4 h incubation in the 3DCFS. Each cell’s intensity profile was analyzed and compared on the vertical and horizontal axis. The actin density was found to be higher in adherent conditions, indicating an elevated stiffness of the cells. Scale bar: 20 µm.

Cell rigidity and deformation are strongly regulated by actomyosin remodeling and contractility [28]. Hence, we examined the role of the cell cytoskeleton by comparing F-actin filament staining of A375 cells in both platforms at the relevant time scales. A375 cells, either adherent or after stirring at 150 rpm for 2 and 4 h, were seeded on coverslips for 24 h and stained with F-actin and DAPI. Confocal imaging revealed a high cell spread (Figure 5a) in the case of the 2D platform and round shape cells (Figure 5b,c) in the case of the 3D platform. The intensity of the fluorescent signal confirmed the broad expression of F-actin filaments in the attached cells (Figure 5a). In contrast, in the floating cells, cortical expression of F-actin together with a high and dense local expression of disorganized F-actin were observed, regardless of the incubation time (Figure 5b,c).

**Figure 6:**
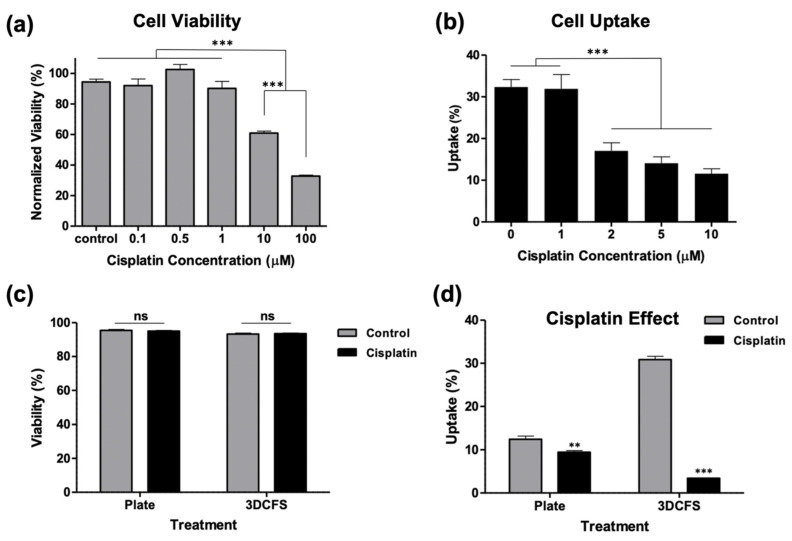
Biochemical manipulation of A375 cells’ mechanics using cisplatin treatment. The A375 cells’ actin structure was reinforced by treating the cells with cisplatin. (**a**) The cells were incubated in 96-well plate for 24 h using different concentration of cisplatin and their viability was measured using WST-1 assay to test which concentration could be used without affecting the cells’ viability. (**b**) The cells were incubated with particles for 4 h in 96-well plate after treatment with different cisplatin concentrations for 6 h. Particle uptake was significantly decreased starting from 2 µM. (**c**,**d**) Cells were treated with 2 µM cisplatin for 6 h and incubated for an additional 4 h both in static and floating conditions and were measured for **c** cells viability and (**d**) particle uptake. *p*-value: *** ≤0.001, ** ≤0.01.

To evaluate the role of cells’ deformability on their ability to uptake particles in adherent and floating conditions, we biochemically manipulated the biomechanical properties of the cells by exposing the cells to cisplatin, which has been shown to significantly increase cell stiffness [29,30]. First, WST-1 viability assay was performed to identify the safe, non-toxic dose. Our results showed that at 1 µM of cisplatin, cells maintained high viability levels, while at 10 µM, their viability significantly decreased compared to non-treated cells (Figure 6a). Based on that, the cells were treated with 1, 2, 5, and 10 µM cisplatin for 6 h and incubated with particles for an additional 4 h. Our analysis showed that treatment with 2 µM cisplatin was the minimal concentration required to affect particle uptake, which resulted in a significant decrease in particle uptake compared to nontreated cells (Figure 6b). The calcein AM viability staining showed that 2 µM cisplatin-treated cells maintained a high viability on both the culture plate and in the 3DCFS after 4 h of incubation (Figure 6c). Importantly, we found that particle uptake was minimally affected by the treatment in adherent cells (~20% reduction) compared to the floating cells that were dramatically affected and reduced the uptake level in 83% (Figure 6d).

## 4. Discussion

Interactions between cells and particles are governed by various chemical, biological and mechanical properties. Parameters such as particle size, elasticity, charge, shape and hydrophobicity determine the interactions with cells, whereas specific cell affinity components may enhance the binding to further increase particle uptake [5,7,9,14,31,32,33]. On the other side, the biological stage of cells (e.g., viability, cell cycle, differentiation) and their biomechanical traits (e.g., elasticity and rigidity) have an enormous effect on particle endocytosis and phagocytosis. Cancer cells of solid tumors are an example of cells that normally adhere to ECM, but the epithelial to mesenchymal transition (EMT) encompasses dynamic changes in the cellular organization from epithelial to mesenchymal phenotypes, and may affect cell functions related to cell migration and invasion. Cancer cells which undergo EMT may detach from their primary mass and colonize in distant organs to form metastasis, as was discussed in detail in a recent review [34]. Normally, the detachment of cells from the extracellular matrix or neighboring cells triggers apoptosis known as anoikis [35,36]; however, malignant cells can often develop a resistance to this cell death and, thus, can survive longer in circulation [36,37]. Little is known about the potential of unanchored cells to uptake particles from their surrounding environment. Therefore, we aimed to study the dependency of cell uptake on their substrate adherence in a systematically and well-controlled system.

Cell uptake assays are typically performed using a planar cell monolayer, while floating systems are predominantly used as bioreactors, using various geometries that ensure cell growth in 3D conditions [38,39]. Our unique 3DCFS device is compatible for uptake assays in floating conditions, and it has the advantage of being designed for standard laboratory equipment; thus, providing a cost-effective and accessible technology.

In this study we used 0.8 µm particles which, based on our previous work [14], assure a deformability mediated uptake. Such a submicron size is ideal for biomechanical studies since smaller particles (<200 nm) use endocytosis which are mediated by specific biological mechanisms (clathrin and caveolin) and bigger particles may adhere rather than internalize into cells in the conditions used in this study.

To ensure cell viability in the 3DCFS apparatus, we monitored A375 cells over 24 h showing a high viability in the first 9 h (Figure 2). Similarly, BXPC-3 (human pancreas) cells under the same conditions confirmed normal rates of cell-proliferated amiability to form new colonies once seeded on a cell culture plate (Figure S3). Despite an equivalent number of cells and particle concentrations in the adherent versus non-adherent experiments, dramatic differences in uptake levels were found. The most extensive particle uptake in the 3DCFS occurred during the first 6 h of incubation while a comparison of the uptake over 4 and 6 h in three types of cancer cells (A375, AsPC3 and MDA-MB 321) revealed a higher uptake in 3DCFS in all cases (Figure 3 and Figure 4). Cell viability, which was substantially low after 24 h in the 3DCFS system (~60% reduction), was probably the main reason for the low particle uptake in the non-adherent system at that time point. To eliminate the cell viability effects and consider biomechanical ones, the of uptake comparative studies were focused for the first 6 h of incubation.

Beyond the effect of cell viability, steric consideration may also have affected the extent of particle uptake. When studying cell spreading using F-actin staining, we detected clear differences in the F-actin filament distribution and expression between glass-adherent cells and cells incubated in the 3DCFS. The non-adherent cells presented peripheral and more diffused and disorganized expression of F-actin after 2 and 4 h of incubation. These results confirmed that dynamical turnover of the cytoskeleton alters actin density, as an adaptation to varying mechanical conditions, in time scale of hours. The differential actin organization suggests that A375 cells undergo cytoskeleton remodeling in suspension and are potentially more deformable than cells grown in adherent and “stretched” conditions. The anchoring of a cell to the ECM is known to involve cell shape changes that produce mechanical stresses on the matrix and in the cell itself [40]. Moreover, the external environment induces the remodeling of the cytoskeleton via changes in actin, as demonstrated in various cells [41,42], while it also induces the adaptation of cell internal elasticity [43]. Interestingly, substrate stiffness was suggested to dictate the behavior of actin cytoskeleton by tuning its rheological properties from fluid-like to solid-like, as the stiffness increases [44]. This might explain the diffused patterns we detected in the cells from the non-adherent source.

In order to study the uptake under the controlled stiffening of cells, we used Cisplatin as a mode for increasing cell rigidity, as previously shown [29], without compromising cell viability. Under these conditions, a high and substantial dependence of particle uptake on floating cells was detected, while a very minor effect was found in the case of the adherent cells (Figure 6). These results indicate the important role of cell deformability on the capacity to execute a rapid uptake of particles in a non-adherence state. The degree of cell uptake was defined in our experiments as the percent of cells loaded with particles out of the total number of cells, according to this fraction:

(1) $$Uptake^{S}\left(t\right)\equiv n_{+}^{S}\left(t\right)/n_{Tot}^{S}\left(t\right)$$

where $n_{+}^{S}\left(t\right)$ represents the number of cells that have one or more internalized particles at time *t*, and

(2) $$n_{Tot}^{S}\left(t\right)=n_{+}^{S}\left(t\right)+n_{-}^{S}\left(t\right)$$

is the total number of cells, with $n_{-}^{S}\left(t\right)$ being the number of cells that did not comprise any particles at time *t*. The superscript *S* stands for the type of the system—either the plate or the 3DCFS.

Importantly, in the case of cells attached to surfaces, we can roughly divide the uptake events into two types: (A) uptake in dividing cells and (B) uptake in non-dividing cells. We found that cell division largely promoted the insertion of particles into the cells (Video S1). This may result from the highly dynamical and irregular state of cell mechanics during cell division, including, for example, the formation of a negative curvature of the cell surface. The time period between dividing events can be approximated as 24 h—the duration at which the number of cells doubles (Figure S1). Thus, the uptake during cell division was expected not to provide a meaningful contribution within the first few hours of the incubation; however, the sharp increase in the fraction of cells that uptake particles in the plate after 24 h (Figure 4) probably results from the rapid cell insertion of particles during division. Cells did not increase in number in the 3DCFS and maintained their viability for up to 9 h (Figure 2 and Figure S1). In suspension, in contrast to 2D conditions, adherent cells generally failed to enter the S phase [45] thus, in the 3DCFS, in contrast to the plate, uptake events probably occurred only by active uptake. In this case, the data could be fitted to an analytical expression, providing that, within an hour, 5.6% of the cells transitioned from not internalizing to internalizing one or more particles (Figure S4).

**Figure 7:**
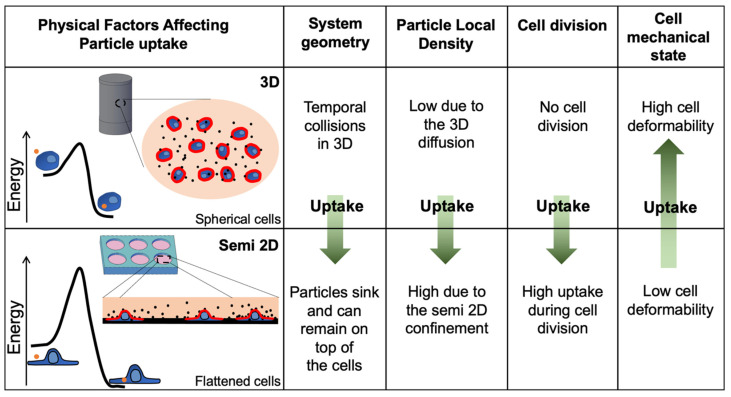
Physical differences between floating and adherent cells affect the uptake kinetics. While several factors favor uptake in the plate, the softness of cells, which favors fast uptake in 3DCFS, is a major term which eventually affects the fast kinetics of particle internalization into the floating cells. The collision rate was expected to be higher in the 2D plate mainly due to the system geometry and to the local density of the particles. In addition, many of the uptake events occurred during cell division while in the 3DCFS, cells did not divide. The observation showing much faster kinetics during the first hours indicated that the major factor was the mechanical flexibility of the cells, which lowered the kinetic barrier in the adhesive interaction between the particles and the cells and allowed for rapid turnover of active deformability of the cells during particle engulfment.

The main physical parameters that can explain the rapid uptake in the 3DCFS relative to the plate, excluding uptake events during cell division, are sketched in Figure 7. Interestingly, many differences between the systems were predicted to contribute to a higher uptake in the semi-2D system, with the exception of cell mechanics. Our clear demonstration of higher and faster uptake in the floating geometry highlights the important role of the mechanical state of the cells in their phagocytic ability. We verified here that cell mechanics is the dominant effect that distinguishes between the systems, by showing that cell stiffening largely reduced the uptake in the 3D system with a negligible effect on cells in the plate (Figure 6).

The neglectable uptake levels measured in time 0 (incubation with particles followed with immediate wash) (Figure 4) suggest that the chance for cell/particle collision in the floating system is not the dominant effector that explains the high uptake levels, since uptake first requires sufficient binding to initiate engulfment as we discussed in detail elsewhere [14].

The rate of uptake depends, on the one hand, on factors related to the frequency of contact with particles and, on the other hand, on the uptake probability once contact occurs [46]. Contact probability is related to collision kinetics. Then, cell–particle interactions are affected by energetic and dynamic (non-equilibrium) factors. Importantly, all these factors depend on the dimensionality of the system.

While suspended cells remain relatively spherical, adherent cells tend to flatten and spread over their culturing substrate [47]. Accordingly, in the 3DCFS device, the exposed surface area that is accessible for particle collisions (red lines in Figure 7) is the complete spherical cell surface, while in the 2D system, the upper facing surface of the flattened cells is predominantly available. This difference was seemingly not significant, since the overall surface area of a spread cell was larger than that of a cell in suspension; however, it cannot be measured precisely. In addition, compared with the 2D system, the particles in the 3DCFS were more dispersed leading to a relative lower particle density in the vicinity of an arbitrary cell, reducing the frequency of cell contact. Moreover, in semi-2D conditions the e-particles can sink and remain on top of the cells for longer periods (“snowflake”-like effect). Another physical argument that favors uptake in the plate relates to particle entropy [48]. The change in particle translational entropy due to cell adhesion was

(3) $$∆S=k_{B}\ln\left(\frac{\Omega_{f}}{\Omega_{i}}\right)$$

where $k_{B}$ is the Boltzmann constant and $\frac{\Omega_{f}}{\Omega_{i}}$ is the ratio between the number of microstates after and before particle absorbance. This ratio was larger in the mixed system, where particles translated in 3D volume and were not restricted to 2D geometry. The higher loss of entropy in the mixed case would yield a lesser uptake than in the 2D system.

Strikingly, while the effects mentioned above favor a rapid uptake in the plate, the experiment results showed a dramatically faster uptake of particles by the floating cells during the first 6 h. Considering the substantially shorter contact time of particles with cells in the floating state, rather than the plate system, the dominant reason for the higher uptake in the 3D system was likely to be the strong effect of cell mechanics. Cells are known to adapt mechanically to their environment and their stiffness increases with the rigidity of the surroundings [49,50,51]. During cell–particle contact, stable quasistatic adhesion is considered a pre-condition for full engulfment and uptake [14]. Then, the active engulfment of the particles can result in a full uptake. Thus, the enhanced cell elasticity and dynamical deformability are essential for the effective internalization of particles into the cells in engulfment procedures. In terms of cell elasticity, for particles that are larger than the mesh size of the cytoskeleton, contact mechanics theories provide a general expression for the dependence of the force *F* on the interaction depth

(4) $$\delta :F\left(\delta \right)=C\left(\alpha ,E,R\right)\cdot \delta^{\alpha }$$

where *E* is the cell Young’s modulus, *R* represents particle dimensions and the exponent α is determined by conditions such as the specific geometry of the interaction [52]. The pre-exponential factor *C* is a monotonically increasing function of *E*. Thus, the energetic penalty in the formation of passive particle absorbance, as represented by $\int F\left(\delta \right)d\delta$, increases with cell rigidity, which is mostly intuitive. Following absorbance, higher dynamic reorganization of the cytoskeleton is expected to complete particle wrapping and penetration into the cell in the floating system. Our results demonstrate a lower density of polymerized actin; this may shift the chemical equilibrium to a higher polymerization. Therefore, in the 3DCFS, a greater cell deformability, whether elastic or dynamic, favors more particle uptake than in the plate.

An additional consideration in particle uptake is the perturbation of the cell surface. Under 3D conditions, the membrane pre-tension was much smaller than in the spread 2D configuration. Thermal fluctuations exert local membrane folds, including convex topologies, which ease particle engulfment. In addition, the large membrane reservoir in the 3D configuration reduces the energy of the membrane expansion that is required for particle engulfment [16,53]. Thus, this factor also favors uptake in the 3D rather than the 2D system.

The considerations related to physical cell shape modulations led to a lower kinetic barrier in particle engulfment by the floating rather than the adherent cells (Figure 7). As a result, cells in the 3DCFS interact and uptake particles much faster than in the plate, signifying the importance of the mechanical considerations while other physical aspects favor the opposite.

Traditional 2D assays, though efficient and practiced, can be biased; at steady-state conditions, particles sink in an arbitrary snowflake-like pattern. Once particles settle, their motion is mostly restricted to the semi-2D geometry, composed of the cell surfaces and the plate bottom. Moreover, adhesion interactions can further inhibit particle mobility. Thus, the traditional approach may not adequately represent the situation in vivo, in which there is always a convection of flow [53] and particles can diffuse in three dimensions. Another important consideration when mimicking physiological conditions is the shear forces under flow. Shear stress was shown to play a substantial role in the tumor metastasis cascade, and to affect parameters such as cell death, proliferation and invasion [54]. The rotation in the 3DCFS imposes azimuthal shear forces on the cells. An examination of particle uptake under a floating condition with opposite blade directions showed a lesser effect in the uptake capability of the cells when the blades were in concave positioning compared to convex positioning. A plausible explanation for that difference may be that the direction of stirring may produce a different shear force, which might also impose mechanical stress on the cells and affect their uptake capacity. This can suggest that although the sheer stress might not be the dominant factor for the elevated uptake under floating conditions, it may still affect and contribute to it. An interesting perspective for the future would be to upgrade the 3DCFS for shear quantification. It should be noted that other potential biological affecters that are sensitive to sheer forces, even if not the dominant parameter in our case, including integrin expression, adhesion complexes, surface molecules, cadherin junctions and more [55,56] may also contribute to the interactions with colloidal systems, mostly in the adherence stage.

## 5. Conclusions

Our observations revealed the major role of cell biomechanics derived from cells’ adherence state, on the cell phagocytosis of inert particles. We found that cell deformability affected the level of particle uptake differently in dependence to the cell adherence state. These significant findings emphasize the need to further explore biomechanically driven mechanisms in the physiological context. Such insights are important for understanding various biological processes and for predicting the potential performance of particulate drug delivery systems. It is yet to be determined how the attachment of immune cells and professional phagocytes is affected by mechanical cues. In this case, molecular factors play a major role; however, similar mechanical trends are expected to be maintained. From the technological aspect, the immense progress in the field of 3D printing enables the development of new, affordable and creative approaches for more precise bioassays.

## Data Availability

Not applicable.

## Supplementary Materials

The following are available online at https://www.mdpi.com/article/10.3390/biomedicines9080947/s1, Figure S1: Cell count under floating and adherent conditions, Figure S2: Cell confluency’s effect on uptake in plate, Figure S3: 3DCFS incubated cell’s recovery, Figure S4: Uptake fitting for particle uptake in the 3DCFS device, Video S1: Time evolution of A375 cells’ 2.4 µm fluorescently tagged polystyrene particle uptake.
