# Supplementary material for: Enhanced Biomechanically Mediated “Phagocytosis” in Detached Tumor Cells
Source: Biomedicines. 2021 Aug 2;9(8):947. doi: 10.3390/biomedicines9080947 (PMC8391740; doi:10.3390/biomedicines9080947)
Supplement: Supplementary file 1 [file biomedicines-09-00947-s001.zip › biomedicines-1309815 -sm.pdf]

Supplementary Information for "Enhanced Biomechanically Mediated "Phagocytosis" in Detached Tumor Cells"

Modification in "magnet above" 3D-printing approach

The initial design of the 3DCFS included a main body with a hinge, blades, and a removable cover, as shown in Figure 1a. The main body was designed as a cylinder with a central hinge and a dome-like tip to facilitate the blade movement. The blade part was designed as a hollow hexagon, with six curved blades tailored to sit on the central hinge of the main body. Above the blades, we placed a 2.3 mm × 6.9 mm chamber for housing a small magnet bar, which, in response to the stirrer, could spin the blades. When tested without liquid, the magnet was able to spin the blades smoothly. However, once 10 mL of water was added to the main container, the magnet was not strong enough to spin the blades due to the resistance caused by the water.

A magnetic field depends on many parameters, such as geometry, media, magnet size and the distance between magnets (in our case the magnet bar and the stirrer's magnet). The media was an unalterable factor, and we did not want to make substantial changes in the geometry. Thus, we decided to change the size of the 3DCFS magnet and to reduce the distance between it and the stirrer, thereby increasing the force between them and enabling the blade's rotation.

In the second version, we enlarged the magnet chamber to 6.05 mm × 20.35 mm to allow a larger magnet (Figure 1b). In this design, the magnet responded to the rotating magnet and spun the blades, but only for a limited time (up to 5 min) even at the maximal rotation velocity. We concluded

that water leaked into the gap between the central hinge and the blades, thus increasing the friction between the parts and inducing resistance in the system.

To resolve this issue, we added an internal cylinder to the main body. The cylinder encircled the central hinge, essentially separating the hinge from the fluid compartment. The blades were adjusted as well, such that the blades' hexagonal core was shortened, while the remaining original 30 mm was replaced by a cylinder. Two outer rings were added to connect and hold the blades (Figure 1c). In this design, the blades operated solely in the fluid compartment of the main body, while ensuring liquid-free contact between the hinge and the blades. When tested with water, the blades spun continuously, without stopping or slowing down. However, when placed inside an incubator with 10 mL of water and stirred at 150 rpm, the system stopped moving after about 1 h. Disassembly of the 3DCFS parts indicated that the upper tip, the "dome," was sticky due to an accumulation of humidity that increased the friction between the central hinge and the blades.

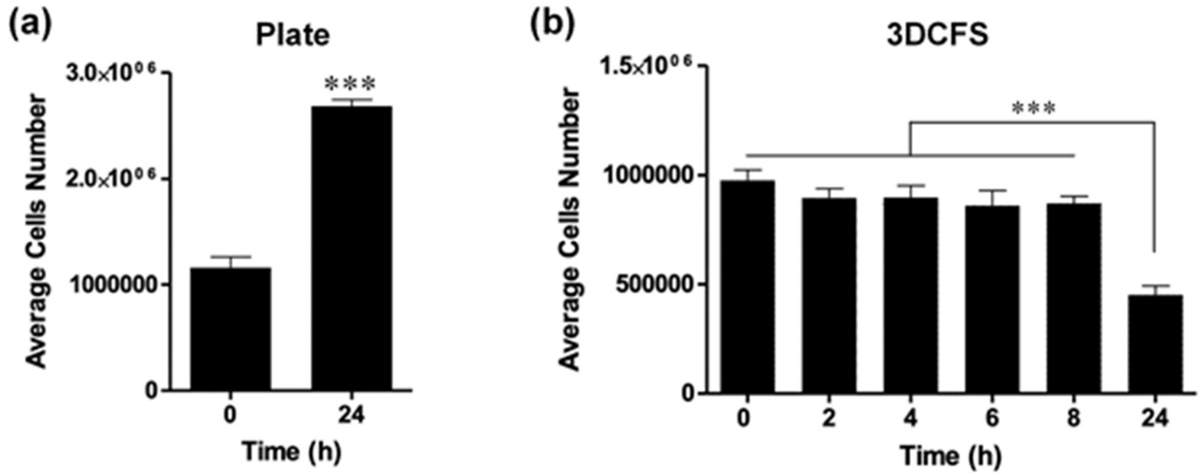

Figure S1. Cell count under floating and adherent conditions. (**a**). Average cell number in a 6-well plate from the moment particles were added (0 h) and after 24 h of incubation. (**b**). A375 cells, together with particles, were added to the 3DCFS for 24 h at a spinning rate of 150 rpm. Cells were sampled and counted after different time intervals. No significant difference was observed during the first 8 h of incubation, while at the 24th hour, the count decreased by 50%. *p*-value: ***≤ 0.001

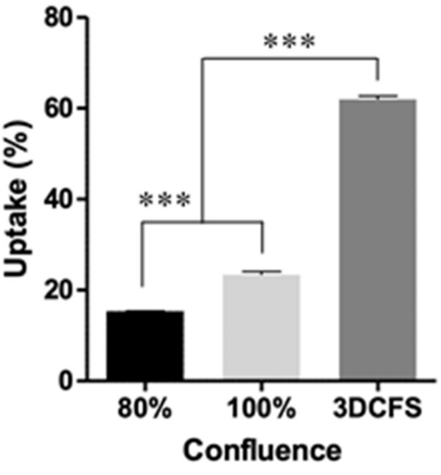

Figure S2. Cell confluency's effect on particle uptake in plate. A375 cells were seeded on a plate to reach 80% and 100% confluency, followed by additional incubation of 4 h with particles. Uptake analysis showed a minor difference in uptake levels between 100% and 80% cell confluency compared to the significant difference between 100% and incubation with particles in the 3DFCS. *p*-value: ***≤ 0.001

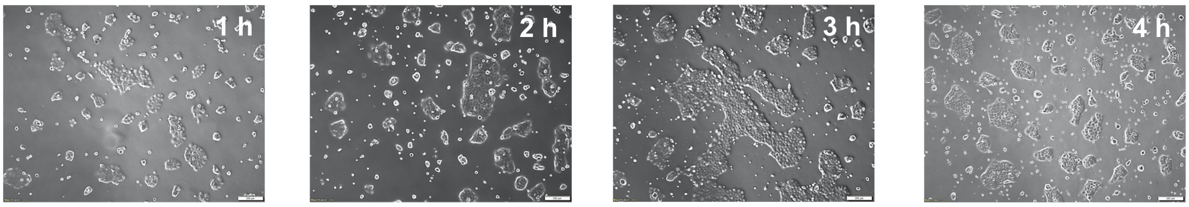

Figure S3. 3DCFS incubated cells' recovery. BXPC-3 cells were added to the 3DCFS for 4 h at a spinning rate of 150 rpm. Cells were sampled at different time intervals, seeded and incubated on 6-well culture plates. They showed formation of colonies after 48 h of incubation. Scale bars = 200 μm.

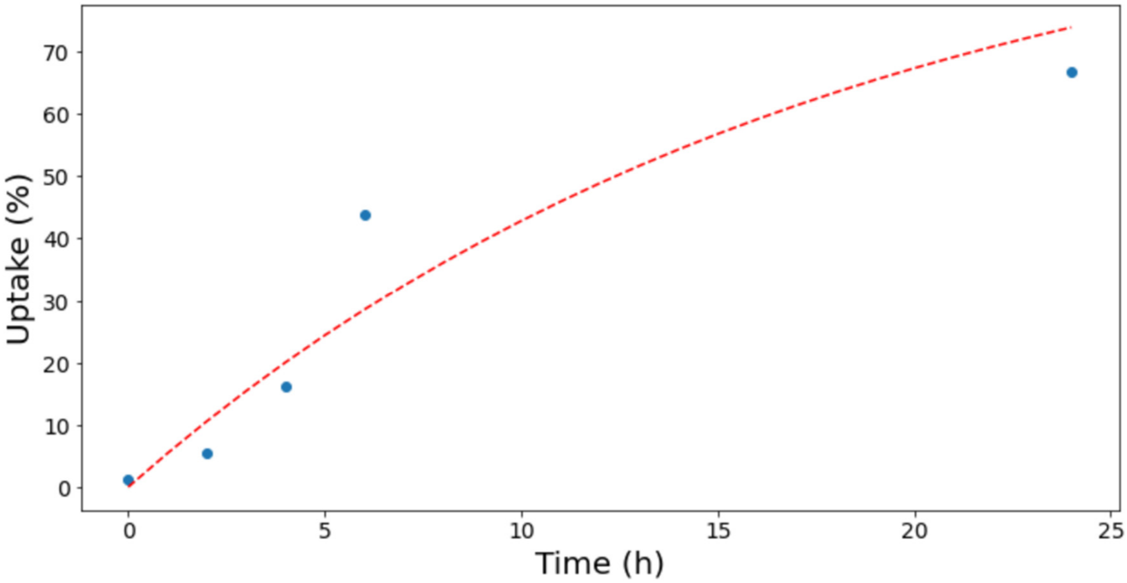

Figure S4. Uptake fitting for particle uptake in the 3DCFS device.

The differential change in the number of cells containing particles can be written in the case of the 3DCFS as:

$$[S1]\ dn_{+}^{3D} = n_{-}^{3D}(t)k^{3D}dt$$

Where $n_{+}^{S}(t)$ and $n_{-}^{S}(t)$ are, respectively, the number of cells that did or did not uptake any particles at time *t*. k^{3D} is the reaction rates per cell not comprising particles, assuming a large concentration of particles. dn_{+}^{3D} is contributed only from the uptake of cells in interphase that previously did not comprise any particles with the rate k^{3D} per cell. Cells that already have particles and uptake more

particles in the time interval $t,\ t+dt$ are not accounted for, as n_{+}^{3D} is the number of cells comprising any number of particles (one or more). Based on our observations, it is assumed here that the exit of particles from cells is negligible (Video S1).

Eq. S1 can be analytically solved assuming that the decrease in n_{Tot}^{3D} is slow, so that $dn_{-}^{3D} = -dn_{+}^{3D}$, providing

$$[2]\ Uptake^{3D}(t) = 1 - e^{-k^{3D}\cdot t}$$

Fitting the uptake data of cells in the 3DCFS with a counterclockwise directionality of the blades is shown in the graph. This fit provided the value of $k^{3D} = 0.056_{1/hr}$, suggesting that 5.6% of the cells transitioned from not containing to containing particles within an hour ($R^2 = 0.89$).

Video S1. Time evolution of A375 cells' 2.4 μm fluorescently tagged polystyrene particle uptake. A375 cells were cultured to ~50% confluency on glass bottom culture dishes (35 mm dish, 14 mm Microwell; MatTek, Ashland, MA, USA). Cells were first exposed to a growth medium lacking serum for 3 h before the experiment and then washed 3 times with HEPES buffer. After the last wash, a HEPES buffer containing 0.1 μM sulforhodamine green (SRG) (Biotium, Hayward, CA, USA) was added to the medium. Cells were imaged with an Olympus FV-1000 confocal microscope equipped with an on-scope incubator (Life Image Services, Basel, Switzerland) with controlled temperature and humidity, and with 5% CO_2. Because the anionic SRG does not enter live cells, the cells appear as dark objects against a uniform fluorescent background when imaged with the confocal microscope. One plane of focus was acquired and then we added the polystyrene particles (time = 0) into the imaging buffer. Images were taken every 5 min over ~12 h and generated a movie sequence. Confocal images of both the SRG (Ex: 514 nm; Em: 535–565 nm) and the polystyrene purple particles (Ex: 560 nm; Em: 600 nm) were acquired from the same section every 10 or 20 sec. The FV1000 confocal microscope was equipped with a Zero Drift Controller option to maintain the same focus plane throughout the entire period of imaging. A ×40 water objective was used.

The movie visualized an increased uptake of particles by dividing cells (demonstrated in the white circles).
